# 

**Published:** 1863-08

**Authors:** 


### CONTENTS.

### ORIGINAL CONTRIBUTIONS.

ART. XIX. MINOR MENTAL MALADIES. By ANDREW McFARLAND, M.D. Read before the Illinois State Medical Society, May 6, and the American Institution for the Insane, May 19, 1863,.................. 353

ART. XX. REPORT OF THE COMMITTEE ON SURGERY, TO THE ILLINOIS STATE MEDICAL SOCIETY. By Professor E. ANDREWS,.................... 369

ART. XXI. TWO CASES OF POISONING BY THE FRUIT OF THE PODOPHYLLUM PELTATUM, OR MAYAPPLE. By Dr. D. C. OWEN, of Houston, Ill., 389

### THE CLINIQUE.

Medical Wards of the Mercy Hospital. Remarks on Diphtheria. By N. S. DAVIS, M.D., Professor of Clinical Medicine, &c., Chicago,............ 392

### BOOK NOTICES

Medical Communications, with the Proceedings of the Seventy-first Annual Convention of the Connecticut Medical Society, held at Rockville, May 27th and 28th, 1863,.................................................. 399

Mortality of Philadelphia for 1862. Report on Meteorology and Epidemics. Read before the College of Physicians of Philadelphia. Feb. 4th, 1863. By Wilson Jewell, M.D., President of the Medical Society of Pennsylvania, &c., &c.,.................................................. 401

Gastrotomy. Large abdominal uterine tumor extirpated by John O'Reilly, M.D., F.R.C.S.I.,.................................................. 481

Lindsay & Blakiston's Physician's Visiting List, Diary, and Book of Engagements,..................................................

Catalogue of Medical, Surgical, Dental, and Scientific Books,.................. 402

The People's Dental Journal. Edited by W. W. Allport, D.D.S., and S. T. Creighton,.................................................. 402

### SELECTIONS.

Mental Condition of the King of Prussia,.................................................. 391

Calomel and Tartar Emetic in the Army,.................................................. 402

The Treatment of Epilepsy by Belladonna. By Dr. J. S. Ramskill, Assistant-Physician to the London Hospital, and Physician to the Hospital for Epilepsy and Paralysis,.................................................. 405

On Scarlet Fever Complicated with Rheumatic and Cardiac Affections,... 409

### EDITORIAL.

Medical Colleges in Chicago,.................................................. 410

Army Surgeons,.................................................. 412

To the Medical Profession of Illinois,.................................................. 412

Illinois State Medical Society,.................................................. 412

Artificial Petrifaction of Animal Tissue,.................................................. 413

Garibaldi Probe,.................................................. 413

Michigan State University,.................................................. 414

Large Scrotal Tumor,.................................................. 414

